# OGG1 Involvement in High Glucose-Mediated Enhancement of Bupivacaine-Induced Oxidative DNA Damage in SH-SY5Y Cells

**DOI:** 10.1155/2015/683197

**Published:** 2015-06-16

**Authors:** Zhong-Jie Liu, Wei Zhao, Qing-Guo Zhang, Le Li, Lu-Ying Lai, Shan Jiang, Shi-Yuan Xu

**Affiliations:** Department of Anesthesiology, Zhujiang Hospital, Southern Medical University, No. 253 Middle Gongye Street, Guangzhou, Guangdong 510282, China

## Abstract

Hyperglycemia can inhibit expression of the 8-oxoG-DNA glycosylase (OGG1) which is one of the key repair enzymes for DNA oxidative damage. The effect of hyperglycemia on OGG1 expression in response to local anesthetics-induced DNA damage is unknown. This study was designed to determine whether high glucose inhibits OGG1 expression and aggravates bupivacaine-induced DNA damage via reactive oxygen species (ROS). SH-SY5Y cells were cultured with or without 50 mM glucose for 8 days before they were treated with 1.5 mM bupivacaine for 24 h. OGG1 expression was measured by quantitative real-time polymerase chain reaction (qRT-PCR) and western blot. ROS was estimated using the redox-sensitive fluorescent dye DCFH-DA. DNA damage was investigated with immunostaining for 8-oxodG and comet assays. OGG1 expression was inhibited in cells exposed to high glucose with concomitant increase in ROS production and more severe DNA damage as compared to control culture conditions, and these changes were further exacerbated by bupivacaine. Treatment with the antioxidant N-acetyl-L-cysteine (NAC) prevented high glucose and bupivacaine mediated increase in ROS production and restored functional expression of OGG1, which lead to attenuated high glucose-mediated exacerbation of bupivacaine neurotoxicity. Our findings indicate that subjects with diabetes may experience more detrimental effects following bupivacaine use.

## 1. Introduction

The risk of severe postoperative neurologic dysfunction is increased in patients with diabetic polyneuropathy undergoing neuraxial anesthesia or analgesia [1], but the mechanism by which high glucose conditions enhance the neurotoxicity of local anesthetics is not fully understood. Clinical trials and basic studies have provided strong evidence that both local anesthetics such as bupivacaine and hyperglycemia can cause neurotoxicity and apoptosis by inducing DNA oxidative damage via enhancing reactive oxygen species (ROS) generation [2–5]. Bupivacaine can uncouple oxidative phosphorylation, inhibit ATP production, and collapse the mitochondrial membrane potential. The decrease in ATP can activate AMPK which results in a marked increase in intracellular ROS [4]. Hyperglycemia can induce ROS overproduction through multiple pathways such as redox imbalances secondary to enhanced aldose reductase activity, altered protein kinase C activity, increased advanced glycation end products, and prostanoid imbalances [6]. Because it results in membrane lipid peroxidation, nitration of proteins, and degradation of DNA, ROS could be an apoptotic trigger in neuronal cell DNA oxidative damage. It is indispensable for the development and progression of neuronal neuropathy, because of the high content of phospholipids and relatively insufficient free-radical defense of nerves [7]. Whether ROS-mediated oxidative DNA damage plays an important role in enhancing the neurotoxicity of the bupivacaine under high glucose condition needs further study to reveal it.

DNA repair pathways are activated to allow damaged cells to survive [8, 9]. Ineffective DNA repair may cause cell apoptosis or disease, which implies that cell fate is influenced by the cell's ability to repair DNA [10]. Apoptosis occurred as a result of irreparable or incompletely repaired genomic DNA, which is constantly subject to assault from intrinsic and environmental insults. ROS are continuously generated as respiration byproducts in mitochondria and are endogenous toxic agents [11]. Oxidized forms of DNA in particular are produced as a byproduct of normal metabolism or in response to exogenous sources of ROS. Base excision repair (BER) pathway is active against much of the damage formed in DNA as a result of cell self-defense mechanism. It is one of the most active DNA repair processes that allows the specific recognition and excision of a damaged DNA base. Oxidative stress is the development of DNA damage, which includes not only a multitude of base modifications, but also base loss and single or double-strand breaks containing sugar fragments or phosphates. All of these lesions are invariably cytotoxic or mutagenic. The majority of damage processed by the BER pathway is generated by the attack of ROS [12, 13]. 8-Oxo-deoxyguanine (8-oxodG) is one of the major base lesions formed after oxidative damage to DNA. 8-OxodG pairs with adenine during DNA synthesis, increasing G:C to T:A transversions. 8-OxodG in DNA is repaired primarily via the DNA base excision repair pathway. The gene encoding the DNA repair enzyme that recognizes and excises 8-oxodG is 8-oxoG-DNA glycosylase (OGG1) [14]. OGG1 deficiency or low expression can limit a cell's ability to repair DNA, leading to the accumulation of DNA damage and eventually to cell apoptosis [15, 16]. Chronic hyperglycemia leads to phosphorylation/inactivation of tuberin and downregulation of OGG1 via a redox-dependent activation of akt, resulting in accumulating cell DNA damage [17]. There is extensive evidence showing that damaged DNA and RNA accumulate in the context of diabetes [18–20]. However, little is known about whether hyperglycemia can aggravate bupivacaine-induced DNA damage and whether hyperglycemia is associated with OGG1 expression in response to DNA damage when diabetic patients receive nerve block anesthesia.

SH-SY5Y cells biological characteristic is similar to normal neural cells. So it is used to research local anesthetic neurotoxicity [2, 4]. We used an in vitro model to investigate OGG1 expression and DNA damage induced by bupivacaine in SH-SY5Y cells treated with high glucose. Our findings may provide a model that is useful for exploring the molecular mechanisms of OGG1 involvement in chronic hyperglycemia-aggravated neurotoxicity in diabetic patients treated with bupivacaine.

## 2. Materials and Methods

### 2.1. Reagents

The human dopaminergic neuroblastoma SH-SY5Y cell line was purchased from the Shanghai Institutes for Biological Sciences. Bupivacaine hydrochloride (purity 99.9%), glucose (purity 99.5%), and N-acetyl-L-cysteine (NAC) were purchased from Sigma (St. Louis, MO). Other reagents used included Dulbecco's modified Eagle medium (DMEM)/F12 (including 17.5 mM glucose) and fetal bovine serum (FBS: Gibco, Grand Island, NY); Cell Counting Kit-8 (CCK8) assay kit (Dojindo, Kumamoto, Japan); 2′,7′-dichlorofluorescein diacetate (DCFH-DA) (Beyotime, China); 4′,6-diamidino-2-phenylindole dihydrochloride n-hydrate (DAPI) which were purchased from Wako Pure Chemical Industries Ltd. (Osaka, Japan); comet assay (Trevigen, Inc., Gaithersburg, MD); anti-OGG1 and anti-8-oxodG (Abcam, Cambridge, UK, ab115841 and ab62623); anti-caspase-9 (CST, USA, 9508) and anti-*β*-actin (KangChen Bio-tech, China, KC-5A08). All reagents were obtained from commercial suppliers and were of standard biochemical quality.

### 2.2. Cell Culture

SHSY-5Y cells were maintained at 37°C in 5% CO_2_ in DMEM/F12 medium, supplemented with 10% FBS and penicillin/streptomycin. Culture medium was renewed once a day during cell growth.

### 2.3. Measurement of Cell Viability

Cells were seeded onto 96-well plates at a concentration of 5 × 10^3^ cells in 200 *μ*L culture medium per well. After serum starvation in DMEM/F12 medium for 24 h, the cells were exposed to 0.5, 1.0, 1.5, 2.0, 2.5, or 3.0 mM bupivacaine for 24 h. Next, 20 *μ*L CCK-8 was added to each well for another 2.5 h at 37°C. Optical density (OD) was read at 450 nm on a spectrophotometer (Bio-Tek, Winooski, VT).

### 2.4. Western Blot Assay

Total proteins were harvested from SH-SY5Y cells with lysis buffer after incubation as described. After centrifugation, protein concentrations were determined by a bicinchoninic acid (BCA) protein assay kit (Beyotime, Haimen, China). Equal amounts of protein (40 *μ*g) were separated by sodium dodecyl sulfate-polyacrylamide gel electrophoresis, electrotransferred to polyvinylidene difluoride (PVDF) membranes, and blocked with 5% nonfat dry milk in Tris-buffered saline. They were then immunoblotted with anti-OGG1 (1 : 500), anti-caspase-9 (1 : 500), or anti-*β*-actin antibody (1 : 1,000) diluted in blocking solution containing 5% nonfat dry milk and 0.1% Tween-20 in Tris-HCl-buffered saline overnight at 4°C. After they were rinsed, membranes were incubated with horseradish peroxidase-conjugated anti-rabbit immunoglobulin at 1 : 1,000 for 1 h. Specific proteins were detected by enhanced chemiluminescence. Finally, the immunocomplexes were visualized using chemiluminescence, and the optical densities of individual bands were quantified using the Chemi-Imager digital imaging system (Alpha Innotech, San Leandro, CA). Band densities were measured using a densitometer and analyzed with Quantity One analysis software (Bio-Rad, Hercules, CA). OGG1 and cleaved caspase-9 protein expression were normalized to their corresponding *β*-actin products.

### 2.5. Quantitative Real-Time PCR (qRT-PCR)

OGG1 mRNA levels were measured by qRT-PCR. After high glucose treatment and bupivacaine administration, total RNA was extracted from SH-SY5Y cells using TRIzol (Invitrogen, Carlsbad, CA, USA). cDNA was synthesized from 2 *μ*g total RNA using PrimeScript RT Master Mix (Takara, Otsu, Japan). Quantitative real-time PCR was performed on a Lightcycler 480 (Applied Biosystems, Foster City, CA) using the SYBR Green Master Mix Kit (Takara, Japan). Relative amounts of OGG1 mRNA were quantified using the 2^−ΔΔCT^ method [21]. qRT-PCR was performed on an ABI Prism 7500 sequence detector (Applied Biosystems, Foster City, CA). The primers were human OGG1 and *β*-actin (OGG1-forward: 5′-CCCTGGCTCAACTGTATCAC-3′; reverse: 5′-TCGCACACCTTGGAATTTC-3′, and *β*-actin-forward: 5′-TGGATCAGCAAGCAGGAGTA-3′; reverse: 5′-TCGGCCACATTGTGAACTTT-3′).

### 2.6. Measurement of ROS

Cells were seeded onto 24-well plates at 5 × 10^5^ cells/well in 500 *μ*L culture medium. Intracellular accumulation of ROS was estimated using the redox-sensitive fluorescent dye DCFH-DA. The cells of each group were incubated with 10 *μ*mol/L DCFH-DA at 37°C during the last 20 min. DCFH-DA-stained cells were washed 3 times in PBS, harvested, and resuspended in PBS. Fluorescence intensity was determined by flow cytometry to estimate relative ROS accumulation.

### 2.7. Immunostaining for 8-OxodG and DAPI

The slices were fixed in 4% paraformaldehyde at 37°C for 30 min. After rinsing with PBS, cells were treated with proteinase K (10 mg/mL) at room temperature for 7 min. After rinsing with PBS, DNA was denatured by treatment with 4 N HCl for 7 min at room temperature. The pH was adjusted with 50 mM Tris-HCl for 5 min at room temperature. After rinsing with 0.2% Triton X in PBS, the cells were stained with a monoclonal anti-8-oxodG (1 : 2000). Alexa Fluor 488-conjugated goat anti-mouse IgG (1 : 200) was used as the secondary antibody. The slices were mounted with Aqua-Poly/Mount (Polysciences, Inc., PA, USA). Fluorescence images were captured using a fluorescence microscope (AX-80, Olympus, Tokyo, Japan), and 20 images per treatment were obtained. The cells immunostained for 8-oxodG were rinsed and then stained with DAPI (2 mg/mL) for 5 min. After rinsing with PBS, the slices were mounted with Aqua-Poly/Mount. Fluorescence images were captured using a fluorescence microscope (AX-80, Olympus, Tokyo, Japan) and merged with 8-oxodG. The positive expression of 8-oxodG was measured by the mean fluorescence intensity using an image analyzer ATTO densitograph (ATTO, Tokyo, Japan).

### 2.8. Comet Assay for DNA Damage

Single cell gel electrophoresis (SCGE), also known as the alkaline comet assay, was used to measure DNA damage [22]. To assess DNA damage products, SH-SY5Y cells were subjected to comet assays. This method measures the ability of damaged DNA to migrate out of the cell when exposed to an electrical field, thus creating a “comet” particle. Undamaged DNA remains in the nucleoid, leaving a spherical particle. Slides were scored for comets using fluorescence microscopy under an inverted microscope (Eclipse TE300, Nikon, Tokyo, Japan) at 200x magnification. Images were captured using Cool SNAPES CCD camera. Fifty cells per slide were analyzed and scored in triplicate using Comet Assay Software Project (CASP) image analysis software (CASP-6.0, University of Wroclaw, Poland). DNA damage is represented as olive tail moment (OTM), which is the product of tail length and percent tail DNA.

### 2.9. Statistical Analysis

Data are presented as means ± standard deviation (SD). Comparisons between two means were performed using independent-sample *t*-tests, and multiple comparisons among groups were analyzed using one-way analysis of variance (ANOVA) with SPSS software 13.0 (SPSS Inc., Chicago, IL). Statistical significance was set at *P* < 0.05.

## 3. Results

### 3.1. The Effect of High Glucose on OGG1 Protein Expression

**Figure 1:**
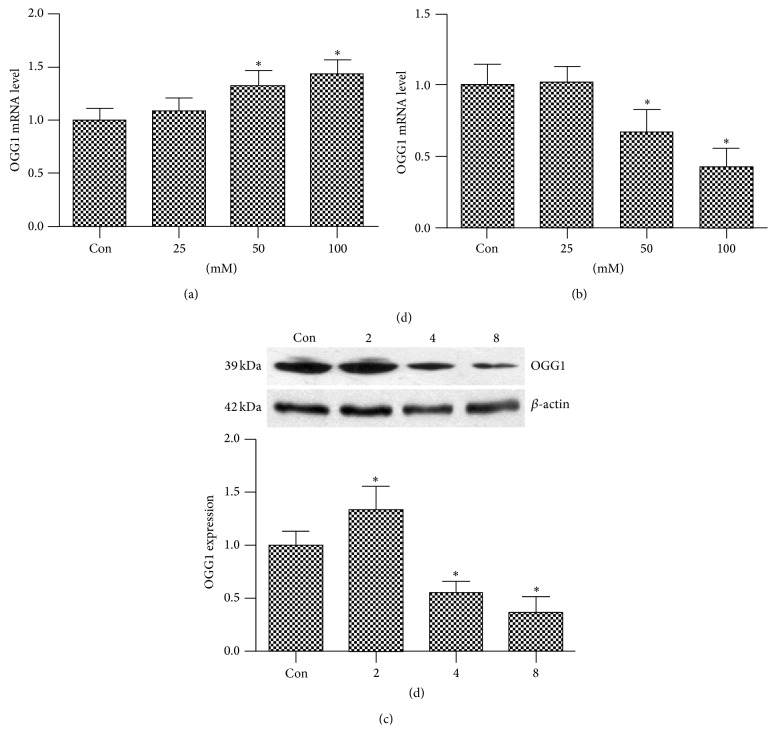
The effect of glucose on the OGG1 expression as detected by qRT-PCR (a, b) and western blot (c). (a) The OGG1 mRNA level in SH-SY5Y cells serum starved for 24 h, followed by incubation with increasing glucose concentrations (25, 50, and 100 mM) for 2 d. (b) The OGG1 mRNA level in SH-SY5Y cells serum starved for 24 h, followed by incubation with increasing glucose concentrations (25, 50, and 100 mM) for 8 d. (c) The OGG1 protein expression in SH-SY5Y cells serum starved for 24 h, followed by incubation with glucose concentration 50 mM for 2, 4, or 8 d. Data are presented as mean ± SD (*n* = 3). Compared with the group Con, ^*∗*^ *P* < 0.05.

We investigated OGG1 mRNA level of SH-SY5Y cells exposed to increasing glucose concentrations (25, 50, and 100 mM) for 2 or 8 days. The result showed that OGG1 mRNA level was associated with concentration and time exposed to high glucose. OGG1 mRNA level was elevated by high glucose at the 2nd day, the higher OGG1 mRNA level when cells exposed to the higher concentration glucose (Figure 1(a)). But OGG1 mRNA level was inhibited by high glucose (50 mM) at the 8th day, the lower OGG1 mRNA level when cells were exposed to the higher concentration glucose (Figure 1(b)). Simultaneously, we investigated OGG1 protein expression of cells exposed to 50 mM glucose for 2, 4, and 8 days and found that OGG1 protein expression was inhibited in a time-dependent manner. OGG1 expression was inhibited by high glucose at the 4th day and onward, with the maximum effect found at 8 d (Figure 1(c)). The results suggested that long-term exposure to high glucose could inhibit OGG1 expression.

### 3.2. Cell Toxicity Induced by Bupivacaine

**Figure 2:**
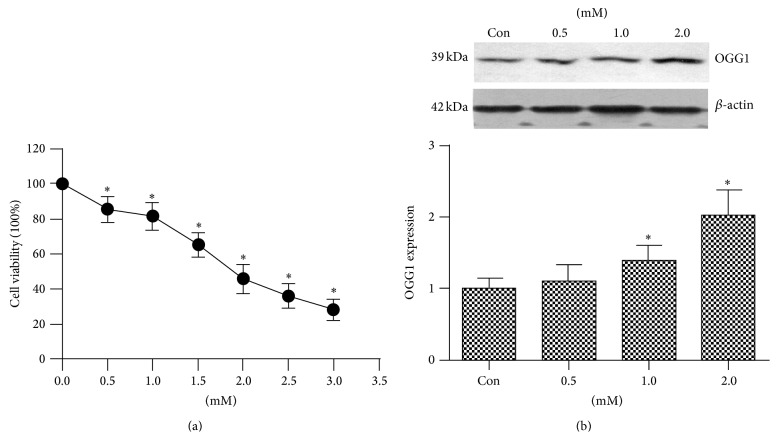
Proliferation effects of bupivacaine on SH-SY5Y cells and the effect of bupivacaine on the OGG1 protein expression. (a) After serum starved DMEM/F12 medium for 24 h, the cell was exposed to 0.5, 1.0, 1.5, 2.0, 2.5, or 3.0 mM bupivacaine for 24 h. Bupivacaine-induced cell injury was detected by CCK8 assay. (b) The OGG1 protein expression in SH-SY5Y cells serum starved for 24 h, followed by incubation with increasing bupivacaine concentrations (0.5, 1.0, and 2.0 mM) for 24 h. Data are presented as mean ± SD (*n* = 3). Compared with the group Con, ^*∗*^ *P* < 0.05.

We compared the cytotoxicity of bupivacaine (0.5, 1.0, 1.5, 2.0, 2.5, and 3.0 mM) using the CCK-8 assay. Our results showed that bupivacaine inhibited cell growth in a concentration-dependent manner. Cell viability was significantly inhibited by 1.5 mM bupivacaine (Figure 2(a)).

### 3.3. The Effect of Bupivacaine on OGG1 Protein Expression

We investigated OGG1 protein expression of SH-SY5Y cells exposed to 0.5, 1.0, or 2.0 mM bupivacaine for 24 h and found that OGG1 protein expression was elevated in a concentration-dependent manner (Figure 2(b)). From the result of cell toxicity, we knew that bupivacaine cytotoxicity was associated with concentration. This result suggested that OGG1 expression could be elevated parallel to increasing concentration of bupivacaine for repairing increasing DNA damage.

### 3.4. High Glucose Inhibited OGG1 mRNA Transcription and Protein Expression in Response to Bupivacaine-Induced DNA Damage

**Figure 3:**
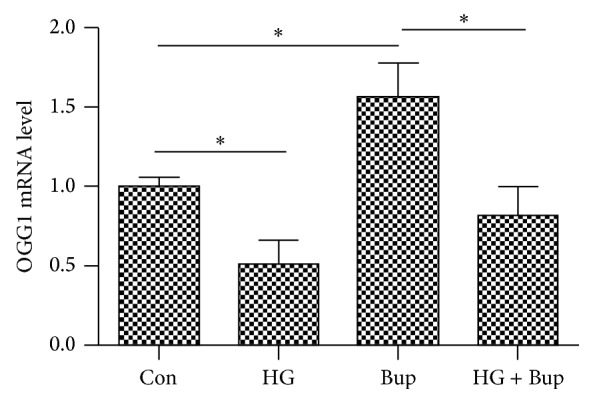
The effect of glucose or bupivacaine on the regulation of OGG1 mRNA level as detected by qRT-PCR. Con: SH-SY5Y cells of group control. HG: SH-SY5Y cells were exposed to 50 mM glucose for 8 d. Bup: SH-SY5Y cells were treated with 1.5 mM bupivacaine for 24 h. HG + Bup: SH-SY5Y cells were incubated with 50 mM glucose for 8 d and then treated with 1.5 mM bupivacaine for 24 h. Data are presented as mean ± SD (*n* = 3). ^*∗*^ *P* < 0.05.

**Figure 4:**
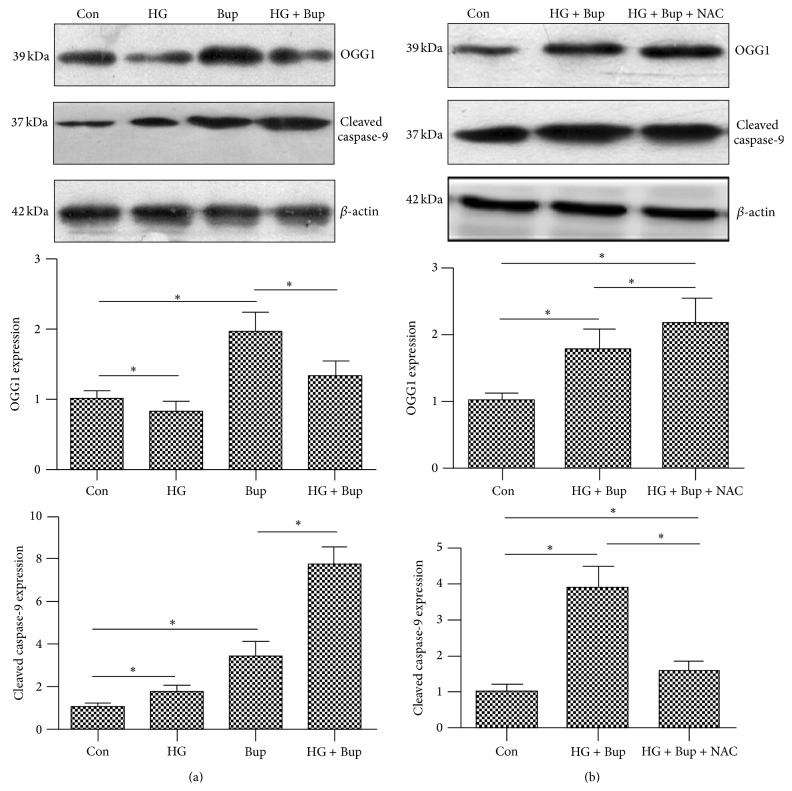
The effect of high glucose, bupivacaine, or NAC on OGG1 and cleaved caspase-9 protein expression as detected by western blot. Con: SH-SY5Y cells of group control. HG: SH-SY5Y cells were exposed to 50 mM glucose for 8 d. Bup: SH-SY5Y cells were treated with 1.5 mM bupivacaine for 24 h. HG + Bup: SH-SY5Y cells were incubated with 50 mM glucose for 8 d and then treated with 1.5 mM bupivacaine for 24 h. HG + Bup + NAC: cells treated with 50 mM glucose for 8 d and then pretreated with 5 mM NAC for 6 h prior to 1.5 mM bupivacaine exposure for 24 h. (a) The effect of high glucose on OGG1 and cleaved caspase-9 protein expression induced by bupivacaine. (b) The effect of NAC on OGG1 and cleaved caspase-9 protein expression induced by high glucose and bupivacaine. Data are presented as mean ± SD (*n* = 3) ^*∗*^ *P* < 0.05.

OGG1 mRNA expression was examined by qRT-PCR and protein expression level was measured by western blot. Compared to the control group, OGG1 mRNA levels and protein expression were reduced in SH-SY5Y cells cultured in 50 mM glucose for 8 d, while they were significantly elevated in SH-SY5Y cells treated with bupivacaine for 24 h. Compared to SH-SY5Y cells treated only with bupivacaine for 24 h, OGG1 mRNA levels and protein expression were significantly reduced in cells treated with high glucose and bupivacaine (Figures 3 and 4(a)).

### 3.5. High Glucose Enhanced ROS Production Induced by Bupivacaine

**Figure 5:**
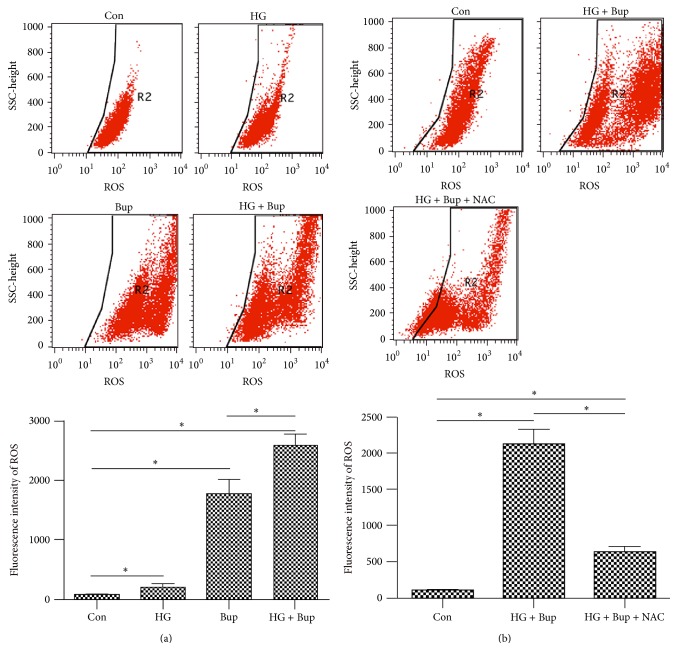
The levels of ROS were measured by flow cytometry. Con: SH-SY5Y cells of group control. HG: SH-SY5Y cells were exposed to 50 mM glucose for 8 d. Bup: SH-SY5Y cells were treated with bupivacaine for 24 h. HG + Bup: SH-SY5Y cells were incubated with 50 mM glucose for 8 d and then treated with bupivacaine for 24 h. HG + Bup + NAC: cells treated with 50 mM high glucose for 8 d and then pretreated with 5 mM NAC for 6 h prior to 1.5 mM bupivacaine exposure for 24 h. (a) The levels of ROS induced by high glucose or bupivacaine. High glucose enhanced ROS overproduction induced by bupivacaine. (b) NAC attenuated ROS overproduction induced by high glucose and bupivacaine. Summarized data shows the fluorescence intensity of ROS as detected by flow cytometry. Data represented are mean ± SD (*n* = 6), ^*∗*^ *P* < 0.05.

Treatment with either 50 mM glucose or 1.5 mM bupivacaine increased the intracellular ROS accumulation, indicated by DCFH-DA fluorescence, while high glucose pretreatment significantly enhanced ROS production induced by bupivacaine (Figure 5(a)).

### 3.6. High Glucose Aggravated Bupivacaine-Induced DNA Damage and Apoptosis

**Figure 6:**
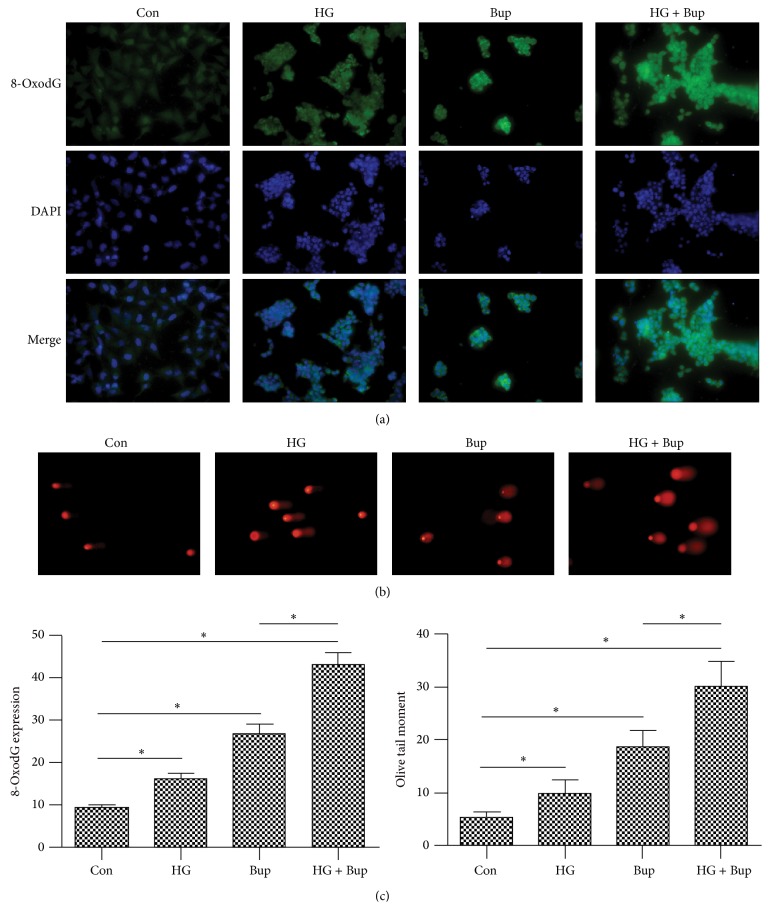
The effect of high glucose or bupivacaine on 8-oxodG expression and DNA damage. Con: SH-SY5Y cells of group control. HG: SH-SY5Y cells were exposed to 50 mM glucose for 8 d. Bup: SH-SY5Y cells were treated with 1.5 mM bupivacaine for 24 h. HG + Bup: SH-SY5Y cells were incubated with 50 mM glucose for 8 d and then treated with 1.5 mM bupivacaine for 24 h. (a) High glucose enhanced 8-oxodG expression induced by bupivacaine. (b) High glucose aggravated DNA damage induced by bupivacaine. (c) Summarized data show 8-oxodG expression as measured by the mean fluorescence intensity and DNA damage as detected by Olive tail moment. Data are presented as mean ± SD (*n* = 3), ^*∗*^ *P* < 0.05.

Oxidative DNA damage product accumulation was quantified by using immunofluorescence detection of 8-oxodG and the OTMs of comet assays. 8-OxodG relative expression and OTM values were increased in SH-SY5Y cells exposed to 50 mM glucose or 1.5 mM bupivacaine and were higher in cells exposed to both agents compared to those only exposed to bupivacaine. These results demonstrate that high glucose enhanced bupivacaine-induced DNA damage (Figure 6).

Cleaved caspase-9 protein expression was measured by western blot. High glucose or bupivacaine treatment resulted in significantly increased cleaved caspase-9 protein levels compared with the control group. Cleaved caspase-9 protein expression was significantly higher in cells treated with high glucose and bupivacaine than in cells treated only with bupivacaine (Figure 4(a)).

Based on the above data, we hypothesized that high glucose could aggravate bupivacaine-induced neurotoxicity in SH-SY5Y cells.

### 3.7. NAC Attenuated DNA Damage Induced by High Glucose and Bupivacaine via Inhibited ROS Production and Increased OGG1 Expression

**Figure 7:**
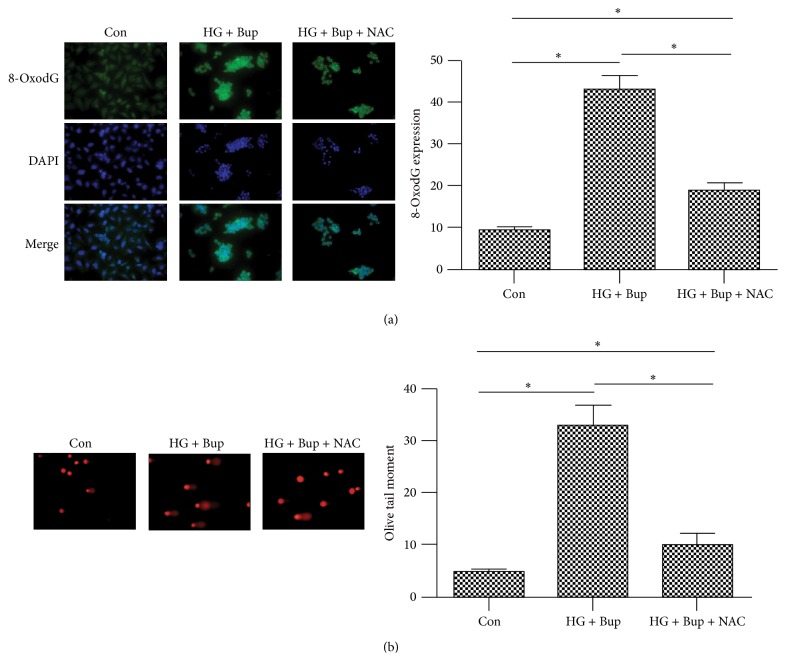
The effect of NAC on 8-oxodG expression and DNA damage induced by high glucose and bupivacaine. Con: SH-SY5Y cells of group control. HG + Bup: SH-SY5Y cells were incubated with 50 mM glucose for 8 d and then treated with bupivacaine for 24 h. HG + Bup + NAC: cells treated with 50 mM high glucose for 8 d and then pretreated with 5 mM NAC for 6 h prior to 1.5 mM bupivacaine exposure for 24 h. (a) NAC attenuated 8-oxodG expression induced by high glucose and bupivacaine. Summarized data show 8-oxodG expression as measured by the mean fluorescence intensity. (b) NAC attenuated DNA damage induced by high glucose and bupivacaine. Summarized data show DNA damage as detected by Olive tail moment. Data are presented as mean ± SD (*n* = 3), ^*∗*^ *P* < 0.05.

NAC pretreatment significantly reduced the ROS overproduction induced by high glucose and bupivacaine and increased OGG1 expression inhibited by high glucose (Figures 5(b) and 4(b)). Simultaneously, NAC attenuated DNA damage and apoptosis induced by high glucose and bupivacaine. However, compared to control group, NAC did not cancel glucose and bupivacaine-induced cell injury (Figures 7 and 4(b)). The above results suggested that high glucose enhanced DNA damage induced by bupivacaine via ROS and NAC could restore functional expression of OGG1.

## 4. Discussion

There are three main findings of the present study. First, long-term exposure to high glucose could inhibit the expression of OGG1 enzyme in SH-SY5Y cells. Second, long-term high glucose exposure inhibited the enhancement of OGG1 expression in response to bupivacaine-induced DNA damage in SH-SY5Y cells. Third, high glucose or bupivacaine can cause DNA damage and apoptosis in SH-SY5Y cells, and these effects were mediated via ROS in cells treated with both high glucose and bupivacaine. Collectively, our findings indicate that high glucose inhibited OGG1 expression in response to DNA damage induced by bupivacaine and aggravated the neurotoxic effects of bupivacaine in SH-SY5Y cells.

Bupivacaine induced depression of the cell respiration related to specific inhibition of complexes I and III, inhibited the production of ATP, and was accompanied with production of ROS. The decrease in ATP can activate AMPK. It can result in a marked increase in intracellular ROS. Overproduction of ROS could result in mitochondrial DNA oxidative damage, caspase activation, and apoptosis [2–4]. This study also showed that bupivacaine exerted concentration-dependent cell toxicity and induced DNA damage and apoptosis via enhancing ROS generation. Hyperglycemia could damage cellular DNA by generating ROS in patients with diabetes [23]. This conclusion is verified by our result showing that high glucose exerted time-dependent toxicity and induced DNA damage and apoptosis via enhancing ROS generation. Under high glucose condition, intracellular ROS production, DNA damage, and cell apoptosis induced by bupivacaine were enhanced. Importantly, cell injury was prevented by antioxidant treatment. It suggested an ROS-mediated mechanism reinforcing the primary oxidative DNA damage of bupivacaine under high glucose condition. Antioxidant treatment may play an important role in preventing and curing hyperglycemia-aggravated neurotoxicity in diabetic patients treated with bupivacaine.

Accumulative ROS could cause DNA damage or mutation. Cells have evolved a diverse defense network to maintain genomic integrity and prevent permanent genetic damage induced by oxidative stress [24]. This results in changes in cellular transcription that, combined with oxidative damage to enzymes involved in processing and repairing DNA damage, might contribute to diabetic neuropathy [14]. 8-OxodG is a sensitive marker of ROS-induced DNA damage [25]. The steady state level of 8-oxodG in DNA reflects its rate of generation and of repairing. 8-OxodG in DNA is repaired primarily via the DNA base excision repair (BER) pathway [26]. One of the key BER enzymes is OGG1, which is necessary for the initial steps in the removal of the mutagenic lesion 8-oxoguanine. Because of its lyase activity, this enzyme may also play a critical role in cleaning the 3-end of oxidative lesions to the sugar-phosphate backbone. The present study showed that bupivacaine could induce ROS overproduction and cause cell DNA damage and apoptosis. 8-OxodG and OGG1 were significantly elevated when cells were exposed to bupivacaine. Bupivacaine-induced ROS resulted in a marked increase in intracellular 8-oxodG. For repairing it, DNA repair enzyme OGG1 was activated, what was cell diverse defense network to maintain genomic integrity and prevent permanent genetic damage induced by oxidative stress. This suggested that BER was involved in repairing bupivacaine-induced DNA damage via ROS in SH-SY5Y cells, and OGG1 might be a crucial factor.

DNA oxidative damage such as 8-oxodG was not effectively removed when OGG1 expression was reduced or inhibited and this could lead to damage accumulation, ultimately resulting in cells aging or apoptosis [27]. Extensive evidence in the literature indicates that diabetes is characterized by the accumulation of damaged DNA and RNA. However it remains unknown whether these lesions are due to inefficient removal by cellular DNA repair pathways [18–20]. Previous study has reported that chronic hyperglycemia could inhibit OGG1 expression via a redox-dependent activation of akt, resulting in accumulating cell DNA damage [17]. This results in deficient DNA repair function and, therefore, leads to the accumulation of DNA lesions. The importance of the OGG1 DNA glycosylase in the repair of oxidative damage was shown in OGG1-deficient mice [28]. These animals accumulate abnormally high levels of 8-oxodG in their genomes. Furthermore, no cleavage of 8-oxoG: C-containing substrate was detected in tissue extracts from OGG1 knockout mice, indicating that OGG1 is the only mammalian glycosylase that can efficiently remove 8-oxodG from 8-oxoG: C pairs. In this study, OGG1 expression was reduced after long-term exposure to high glucose and then this leads to accumulating 8-oxodG. This result suggested that chronic hyperglycemia could result in DNA damage that overwhelmed the DNA repair defense system, which may induce genomic instability and cell dysfunction. Our study showed that SH-SY5Y cells cultured with high glucose and bupivacaine decreased OGG1 expression and resulted in more severe DNA damage and apoptosis. Under high glucose condition, OGG1 expression was inhibited, and it could not effectively repair increasing 8-oxodG induced by bupivacaine and high glucose. This leads to damage accumulation, ultimately resulting in cells apoptosis. NAC could restore functional expression of OGG1 inhibited by high glucose and attenuate this damage. It suggested that antioxidant could reverse hyperglycemia-induced inhibitory effect on OGG1. However, the way of NAC promoting OGG1 expression under high glucose condition remains unknown and needs further research to reveal it. This result demonstrated that under high glucose environment, the decrease in OGG1 was involved in enhanced DNA damage and apoptosis induced by bupivacaine. Antioxidant therapy could reverse these deleterious effects in part by restoring function of the DNA repair enzyme OGG1.

Some limitations of this study should be noted. First, we did not investigate the mechanism underlying the effect of bupivacaine on OGG1 in SH-SY5Y cells cultured in high glucose. Second, the bupivacaine concentration used in this study was 1.5 mM, which is equal to 0.045%. Local injection concentrations are generally 0.5% or 0.75%. So, this dosage is not precisely clinically relevant. Bupivacaine concentration-dependent cell toxicity did not allow us to determine whether bupivacaine at higher clinical concentration would kill all cells. However, in spinal or epidural anesthesia, the axons in the nerve roots of the cauda equina bear the brunt of the high initial bupivacaine concentration. So, the result of the study suggests that diabetic patients treated with bupivacaine may suffer from nerve or neuronal damage. Third, we did not perform in vivo experiments to validate our conclusions.

In conclusion, high glucose could inhibit OGG1 expression in response to bupivacaine-induced DNA damage and simultaneously aggravate the neurotoxic effects of bupivacaine via ROS in SH-SY5Y cells.

## Abbreviations

OGG1: 8-OxoG-DNA glycosylase
8-oxodG: 8-Oxo-deoxyguanine
ROS: Reactive oxygen species
NAC: N-Acetyl-L-cysteine
OTM: Olive tail moment.
